# The Variability of Nitrogen Forms in Soils Due to Traditional and Precision Agriculture: Case Studies in Poland

**DOI:** 10.3390/ijerph18020465

**Published:** 2021-01-08

**Authors:** Anna Podlasek, Eugeniusz Koda, Magdalena Daria Vaverková

**Affiliations:** 1 Department of Revitalization and Architecture, Institute of Civil Engineering, Warsaw University of Life Sciences, Nowoursynowska 159 St., 02-776 Warsaw, Poland; eugeniusz_koda@sggw.edu.pl (E.K.); magda.vaverkova@uake.cz (M.D.V.); 2 Department of Applied and Landscape Ecology, Faculty of AgriSciences, Mendel University in Brno, Zemědělská 1, 613 00 Brno, Czech Republic

**Keywords:** ammonium, nitrate, migration of nitrogen forms, fertilization

## Abstract

The soil and human health issues are closely linked. Properly managed nitrogen (N) does not endanger human health and increases crop production, nevertheless when overused and uncontrolled, can contribute to side effects. This research was intended to highlight that there is a need for carrying out monitoring studies in agricultural areas in order to expand the available knowledge on the content of N forms in agricultural lands and proper management in farming practice. The impact of two types of fertilization, concerning spatially variable (VRA) and uniform (UNI) N dose, on the distribution of N forms in soils was analyzed. The analysis was performed on the basis of soil monitoring data from agricultural fields located in three different experimental sites in Poland. The analyses performed at selected sites were supported by statistical evaluation and recognition of spatial diversification of N forms in soil. It was revealed that the movement of unused N forms to deeper parts of the soil, and therefore to the groundwater system, is more limited due to VRA fertilization. Finally, it was also concluded that the management in agricultural practice should be based on the prediction of spatial variability of soil properties that allow to ensure proper application of N fertilizers, resulting in the reduction of possible N losses.

## 1. Introduction

Excess nitrogen (N) in the soil, aquatic and atmospheric environments is a global problem, resulting mostly from human activities, which have a great impact on the N cycle at all environmental scales [1]. Available N from soil, fertilizers, and manure sources, when inefficiently used in crop production systems, can move from agricultural fields and contaminate surface and groundwater resources and also contribute to greenhouse gas emissions [2]. 

In recent years, numerous technologies and scientific attempts have been employed to improve the efficiency of N use. New technologies are especially intended to predict and control the N impact on the natural environment. They are also crucial for accurate measurement of spatial variability of crop yields and N availability in soils. Several authors reported that the most significant factors that determine N content in soils are, i.e., bulk density, soil clay content, organic matter, pH, climate, vegetation, terrain topography, and human activity [3,4]. The amount of N released into the soil–water environment is also significantly influenced by factors like the dose and type of N fertilizers, time and frequency of their application, efficiency of the use of N by plants, depth of the plant root system, and soil permeability [5,6,7,8]. According to Mosier et al. [9], in Europe, where N fertilizers are used the most frequently, leaching and denitrification are the main processes responsible for N losses to the environment. 

The nitrate and ammonium ions released in the solubilization process are used by plants and affect crop yield. However, their excess (especially nitrate) may penetrate into groundwater. Ammonium ions supplied in mineral fertilizers (e.g., ammonium nitrate), despite the fact that they are not susceptible to leaching, like nitrate ions, may contribute directly to soil acidification. Increasing the content of nitrite (NO_2_^−^) and nitrate (NO_3_^−^) in plants is also treated as an undesirable phenomenon due to the harmfulness of N compounds to animal and human organisms. For example, N compounds, including nitrate and nitrite, were classified along with lead (Pb), cadmium (Cd), and sulfur (S), among the most dangerous factors that can have a harmful effect on human and animal health. The ammonium can also undergo a nitrification process carried by *Nitrosomonas* and *Nitrobacter*, contributing then to the accumulation and further loss of nitrate.

N losses associated with its leaching can vary significantly even over short distances of the agricultural lands, due to the variability of the soil profiles and landscape topography. These differences may also be visible in specific regions where different fertilization practices are applied. It is also possible to distinguish temporary changes in the amount of leached N forms, which is related to the climatic conditions. Gray [10] reported that in favorable environmental conditions, about 50–70% N supplied in fertilizers could be taken up by plants, 2–20% of the delivered dose is lost to the atmosphere, 15–25% is retained on organic particles and clay minerals, and 10% is leached to groundwater and surface waters. Leaching of inorganic N forms from arable fields takes place by gradual displacement of nitrate to deeper layers of the soil. Füleky [11] assessed that, on average, nitrate leaching losses were equal to 30–40 kg ha^−1^ from sandy soils and 20–30 kg N ha^−1^ from loamy soils, whereas the N dose applied during the treatment was in the range 112–280 kg N ha^−1^ year^−1^. 

Therefore, in relation to the above, it is necessary to take actions to reduce the concentration of nitrate in groundwater and surface waters in arable lands as well as to monitor the N cycle in the soil and aquifer in order to assess the impact of fertilization on the quality of the soil–water environment, and consequently on human health [12,13,14]. 

The common agricultural policy (CAP) was launched in the European Union (EU) as a partnership between agriculture and society, as well as between Europe and its farmers. The CAP is intended to: support farmers and improve agricultural productivity; to make reasonable living for EU farmers; to help tackle climate change and the sustainable management of natural resources; to maintain rural areas and landscapes across the EU; and to keep the rural economy alive by promoting jobs in farming and associated sectors. The second pillar of CAP is rural development, which aim is providing member states with an envelope of EU funding to manage nationally or regionally under multiannual, co-funded programs. The Polish Rural Development Program (RDP) is aimed at all six rural development priorities with the main priority being farm viability and competitiveness. The RDP focuses on the support of physical investments as well as on farms located in nitrate vulnerable zones and Natura 2000 areas. In addition, the support is reserved for environmental and climate friendly services and practices aimed at enhancing biodiversity, high nature value farming, improving water management, and preventing soil erosion. The focus of adopted priorities concerns: (1) Knowledge transfer and advisory services in agriculture; (2) Competitiveness of agricultural sector; (3) Food chain organization, including processing and marketing of agricultural products, (4) Restoring, preserving, and enhancing ecosystems; (5) Low carbon and climate resilience economy in the agriculture and forestry sector; (6) Social inclusion and local development.

In Poland, within 14.5 million hectares of utilized agricultural area, 74.7% is arable land and 22.4% is permanent grassland and meadows. The total population is 38.5 million and there is a relatively high share of the population working in agriculture (12%, compared to the EU average of 5%) due to the socio-economic structure of Polish agriculture which is dominated by small family farms (out of 1.5 million farms, c.a. 55% are below 5 ha). Moreover, low soil quality, combined with frequent rainfall shortages, also have a negative impact on agricultural productivity. Due to that, the 62.5% of agricultural land is classified as areas with natural constraints (ANC) [15]. Approximately 19.4% of arable lands in Poland face various environmental challenges. It concerns areas particularly endangered by water and/or wind erosion (8.2%), problems with low humus levels (3.6%), and nitrate vulnerable zones (7.4%) [15]. The relatively intensive use of arable land is putting pressure on ecosystems and naturally valuable areas. Moreover, the relatively poor water quality and the high eutrophication of Polish lakes, waterways, and the Baltic Sea make it necessary to reduce nitrogen, phosphorus, pesticide, and herbicide emissions.

**Figure 1:**
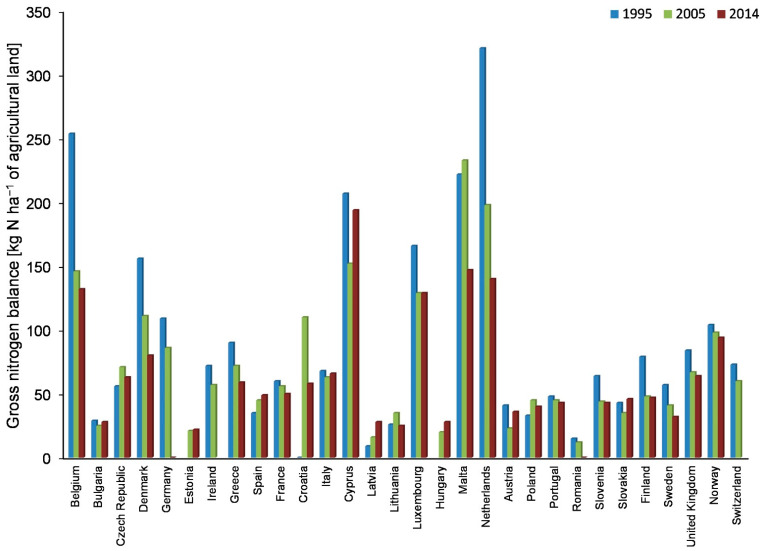
Annual gross N balance (kg N ha^−1^ of agricultural land) in selected EU countries (own study based on Eurostat data [15].

In EU countries, significant changes are observed with regard to reducing the use of fertilizers and thereby reducing the N balance surplus, leading to a more efficient use of this nutrient in agriculture. In the 1990s, the annual N surplus reached over 200 kg N ha^−1^ for agricultural lands in Belgium, Malta, and Cyprus, and even up to 300 kg N ha^−1^ for agricultural lands in the Netherlands. Currently, the largest annual N surplus, exceeding 100 kg N ha^−1^, is still observed in the abovementioned countries. Poland, with the annual surplus of 40 kg N ha^−1^, belongs to the group of EU countries with the lowest gross N balance (Figure 1). 

The losses of fertilizer components (i.e., N forms) from agriculture can be reduced by using modern fertilization techniques within precision agriculture (Table 1). The principles of precision fertilization are most often associated with limiting the excessive application of mineral fertilizers and reducing the risk of losses of unused fertilizer components. The most important approach is the optimization of fertilization rates, regarding the method and time of fertilizer application, adapted to the current needs of plants.

Moreover, the application of spatially variable (VRA) rates of fertilizers improves soil productivity and the efficiency of fertilization [37]. It is also advisable to divide the total N dose into several splits applied on several dates, during the period of plant growth, due to the fact that a high single dose of easily soluble mineral fertilizers causes the accumulation of N in the soil and as a result can lead to groundwater pollution. As an example, Kabala et al. [38] reported that a single application of the entire fertilizer dose (90 and 180 kg ha^−1^) resulted in a significantly higher concentration of ammonium and nitrate in soil compared to a split dose. The authors also confirmed that the concentrations of nitrate and ammonium in soil were the highest with standard urea fertilization and the lowest in variants fertilized with slow-release urea. It was also pointed out that higher concentrations of both N forms were noted at the fertilizer dose of 180 kg ha^−1^. Findings presented in the cited study indicated that the concentrations of N forms in soil are influenced by the type of fertilizer, fertilizer dose, and its division into parts.

The determination of soil mineral N patterns and variability is extremely important for agricultural management and planning [39,40]. Knowledge about the N content in the soil is also important for hydrogeological modeling and predictive analysis of N fate and transport [41,42]. Continuous development of modeling techniques and the widespread use of computational programs make it possible to solve most of the environmental tasks, including estimation of the extent of pollutant or nutrients migration. Nevertheless, it involves the risk connected with the lack of sufficient data concerning parameters occurring in mathematical equations describing the processes of pollutant transport. Typically, the prediction of contaminant migration parameters is based on the data found in the literature without considering under what conditions they were measured. Hence, the basis for creating each model of migration of N forms is always a proper database with the parameters of the environment in which the migration or transformation takes place [43]. 

Our study was intended to highlight that there is a need for carrying out monitoring studies in agricultural areas in order to expand the available knowledge on the content of N forms (the range and spatial diversification) in agricultural lands of selected experimental sites in Poland. We hypothesized that the concentration and distribution of N forms in soil is impacted by the fertilization method as well as the content of N forms in soil is spatially diversified and correlated with the content of clay, silt, and sand fractions. Due to the above, the objectives of this study were: (1) to revise the literature findings in aspect of N forms content in soils and factors influencing them, and (2) to evaluate the effect of UNI and VRA N application as well as soil texture on the variability of N forms in soils.

## 2. Materials and Methods 

### 2.1. Study Site Description and Soil Sampling

**Figure 2:**
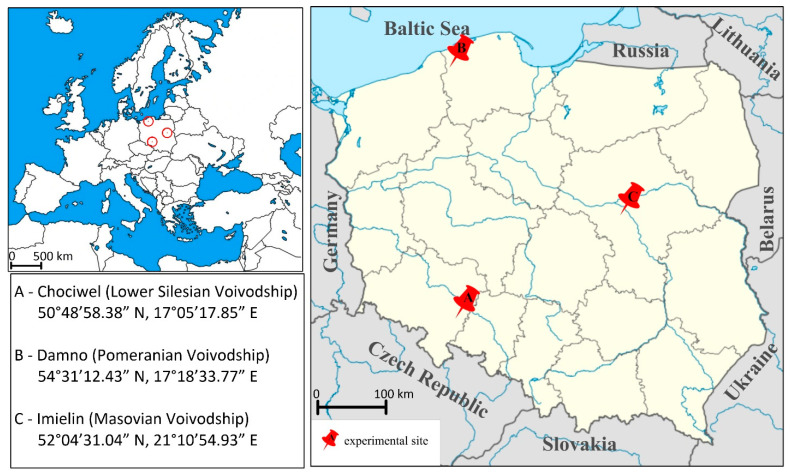
Locations of experimental sites.

Soil samples were collected from three agricultural fields in Poland, located in the Lower Silesian (A—50°48′58.38″ N, 17°05′17.85″ E), Pomeranian (B—54°31′12.43″ N, 17°18′33.77″ E), and Masovian Voivodships (C—52°04′31.04″ N, 21°10′54.93″ E), respectively (Figure 2). 

**Figure 3:**
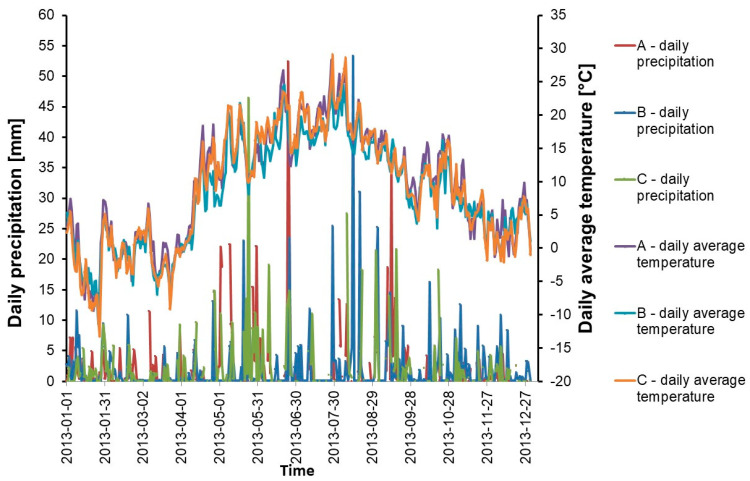
Meteorological data for the A, B, C experimental sites in the year of the research: A−Chociwel in Lower Silesian Voivodship; B−Damno in Pomeranian Voivodship, C−Imielin in Masovian Voivodship.

The selected study sites are comparable by means of meteorological conditions (Figure 3), described by the data obtained from meteorological stations located nearby the experimental sites. 

The average concentrations of N forms in soil before the application of N fertilizers were as follows:

- A experimental site: 1.1 mg kg^−1^ (NH_4_-N), 4.3 mg kg^−1^ (NO_3_-N), 0.4 g kg^−1^ (N_Kjeldahl_);
- B experimental site: 0.9 mg kg^−1^ (NH_4_-N), 10.2 mg kg^−1^ (NO_3_-N), 0.8 g kg^−1^ (N_Kjeldahl_);
- C experimental site: 1.9 mg kg^−1^ (NH_4_-N), 15.1 mg kg^−1^ (NO_3_-N), 0.6 g kg^−1^ (N_Kjeldahl_).

In this research, the analyzed sites were fertilized with two different method (Table 2). 

The first one considered traditional fertilization with the use of uniform N dose of fertilizer for the entire field. The second one concerned precision fertilization with the use of spatially variable rate of N fertilizer. In the case of precise fertilization, the N rate was adjusted to plant demands on the basis of measurement performed by the use of Veris mapping system which allows for the rapid determination of pH, electrical conductivity, and the content of organic matter in soil.

During the monitoring period, winter wheat was cultivated at tested sites. The locations of the sampling points were designated with the density of approximately one soil sample per one hectare. The co-ordinates of sampling points were set in reference to the PUWG 1992 geodetic system using the GPS equipment. The number of soil samples collected for laboratory analyses of N form concentrations varied from 18 to 39. To track the content of N forms in the topsoil, the soil samples were taken from the depths of: 0.00–0.30 m; 0.30–0.60 m, and 0.60–0.90 m. The particle size analysis of soil samples was performed using the sieve and areometric methods according to PN-B-04481:1988 [45]. The basis of a sieve analysis was to allow the non-cohesive soil to pass through a series of sieves of progressively smaller mesh size and weighting the amount of material that was stopped by each sieve as a fraction of the whole mass. Then, the percentage of grains remaining on the sieves was calculated in relation to the total mass of the tested sample. The areometric method was based on determination of the falling speed of soil particles in water. During the examination, the change in suspension density over time was evaluated with the use of the areometer. By means of areometric analysis, the actual dimensions of the soil particles were not determined, but the equivalent diameters, i.e., the diameters of the particles with the same specific density of the soil skeleton as the tested soil and falling in water at the same speed as real particles.

Soils were classified in accordance with the U.S. Department of Agriculture [46]. Soil particle-size distribution was described in relation to the percentages of sand (Sa) (0.05–2.0 mm), silt (Si) (0.002–0.05 mm), and clay (Cl) (<0.002 mm). 

### 2.2. Laboratory Analysis

The ammonium content in soil was determined by a colorimetric method with Nessler’s reagent, whereas the nitrate content was determined by a colorimetric method with phenol disulfonic acid, using a UV-VIS DR 6000 spectrophotometer. In this method, ammonium and nitrate ions were extracted from the soil using 1% K_2_SO_4_ solution. The sum of ammonium and organic N (N_Kjeldahl_) in soil was determined in accordance with application note ASN 3313 [47].

### 2.3. Statistical Calculations

The results of laboratory tests were subjected to statistical analysis using Statistica 12 software (StatSoft Inc., Tulsa, OK, USA). The data set was tested for normality using the Shapiro–Wilk test. In case of a lack of normality, the Mann–Whitney U test [48] was applied to check the differences between measured variables (N concentrations). The homogeneity of variances was checked using the Levene test. When the data revealed the normality, the Student *t*-test was used [49]. The significance of differences in ammonium, nitrate, and the sum of ammonium and organic N were checked for both uniform and spatially variable (UNI and VRA) fertilization systems. It was assumed that the average concentrations of N forms are the same after UNI and VRA variable fertilization (c_1_ = c_2_, where c_1_—average concentration of N form after UNI fertilization, c_2_—average concentration of N form after VRA fertilization). The differences in the concentrations of N forms at the examined sites after UNI and VRA fertilization were also compared. The hypothesis was based on the assumption that the concentrations of N forms in soils after UNI or VRA fertilization do not differ significantly between the analyzed fields. A comparative analysis of the N content at different sites after different types of fertilization was also performed following the Kruskal–Wallis test [50]. To check whether our statistical testing was performed properly, we have also used the post-hoc tests. The post-hoc tests were performed to refine the differences detected by analysis of variance. This allowed the separation of homogeneous groups. Two tests were used: Duncan’s test and Fisher’s NIR test, which are usually used as verification for other tests. Performed tests allowed us to distinguish one or two homogenous groups. The Spearman correlation coefficients were calculated to determine the relationships between the concentration of N in soils and various soil properties. Moreover, the distribution maps of the content of N forms in the soil were prepared using Surfer 10 software. The soil data were interpolated using the kriging method [51], which is commonly used in order to estimate the spatial distributions of pollutants, nutrients, or other soil properties.

## 3. Results

### 3.1. Distribution of Soil Types

**Figure 4:**
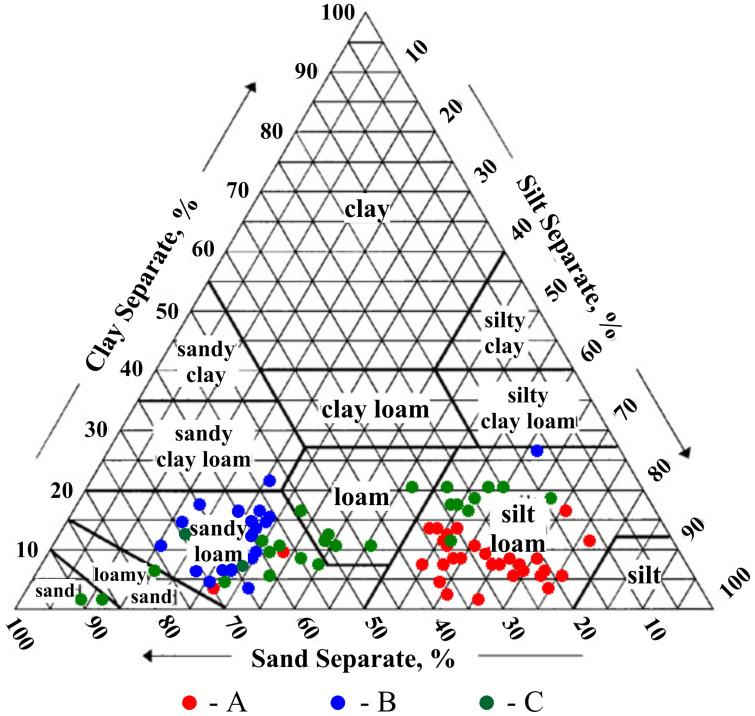
Classification of soils collected from the analyzed experimental sites A, B, C.

The performed investigations revealed that silt loams dominate in the soil at the A experimental site (Chociwel in Lower Silesian Voivodship) (Figure 4). 

The average content of sand, silt, and clay fractions in soils collected from this experimental field were: 32%, 60%, and 8%, respectively. Soils collected from the B experimental site (Damno in Pomeranian Voivodship) were represented mainly by sandy loams and contain on average 63% sand, 26% silt, and 11% clay fractions. Soils from the C experimental site (Imielin in Masovian Voivodship) were characterized by the greatest variability. In majority, they were represented by sandy loams, loams, and silt loams. The average contents of sand, silt, and clay fractions in soils collected from this site were: 48%, 40%, and 12%, respectively. 

### 3.2. Statistical Testing of Differences between N Forms Content in Soil

Based on the results of the Mann–Whitney U test (Table 3), the concentrations of ammonium after UNI and VRA fertilization were different at experimental sites A and B. 

Like the concentration of ammonium in soils, the concentration of nitrate was different between both types of fertilization at the experimental site A. The homogeneity of variances was checked using the Levene test (*p* = 0.119959), and the obtained significance level meant that the variances were homogeneous. Moreover, the N_Kjeldahl_ was found to be significantly different for both fertilization strategies at the A and C experimental sites. 

The calculations made by the Kruskal–Wallis test indicated that the experimental sites A, B, and C did not differ significantly (*p* = 0.0745) with regard to ammonium concentration when UNI fertilization was applied. Based on the results obtained, it was also revealed that experimental sites differ significantly (*p* = 0.0000) in terms of nitrate in soils when UNI fertilization was applied. The same conclusion can be drawn when comparing the differences of the sum of ammonium and organic N in soils taken from the experimental sites after UNI fertilization (*p* = 0.0000). 

The analysis performed within the presented study showed that the sites differ significantly in terms of ammonium concentrations when VRA fertilization was adopted (*p* = 0.0002). In contrast to the above, the concentrations of nitrate and the sum of ammonium and organic N in soils did not differ significantly at the analyzed fields after VRA fertilization, which was proven by the Kruskal–Wallis test (*p* = 0.0654 for N-NO_3_^−^ and *p* = 0.1432 for the sum of ammonium and organic N). 

It was revealed by the post-hoc tests that the object C was different from the object A and B, by means of ammonium concentration after UNI fertilization. It was also indicated that the objects B and C are similar by means of ammonium concentration after VRA fertilization and nitrate concentration after UNI concentration. All of tested objects were similar by means of nitrate concentration and the sum of ammonium and organic N after VRA fertilization. The post-hoc tests allow us also to draw the conclusion that the sum of ammonium and organic N at the B experimental site is different from the concentrations measured at A and C experimental sites.

In this study, we based our approach, and then the interpretation of results, on findings presented in guideline adopted for Statistica software, reported by Rabiej [49] who presented several examples which indicate that the interpretation of data (and its significance) should come down to *p* value analysis (*p* greater or lower than *α*). Nevertheless, the researchers should be careful when interpreting their data based on only *p* values and all results should be interpreted in the context of the study design, including the nature of the samples, the concept of the experiment, validity of instruments, and rules with which the study was performed.

### 3.3. Content of N Forms in Soil vs. Soil Depth

The results show that the concentrations of N forms in the soil profile were negatively correlated with the soil depth for both types of fertilization (Table 4). 

**Figure 5:**
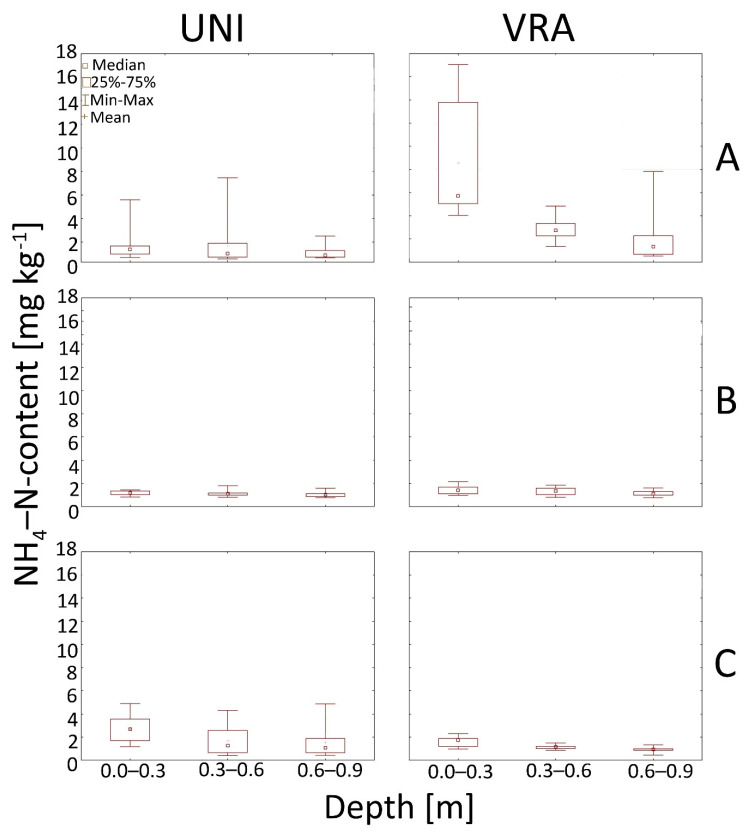
Box and whiskers plots presenting the ammonium content in the soil after UNI and VRA fertilization at the analyzed experimental sites A, B, and C. (**A**–**C**).

It was also shown that greater concentrations occurred after VRA fertilization at A and B experimental sites (Figure 5). 

The highest concentrations of NH_4_-N in soils were observed after VRA fertilization at the A experimental site. The average concentrations of ammonium in the soil at the B and C experimental site were slightly lower and did not exceed 3 mg kg^−1^.

**Figure 6:**
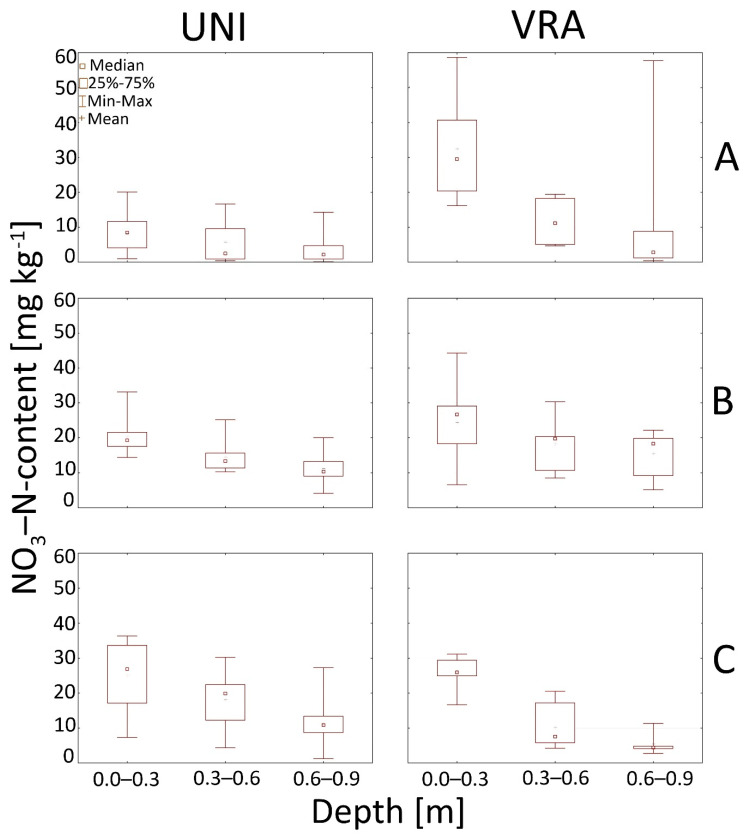
Box and whiskers plots presenting nitrate content in the soil after UNI and VRA fertilization at the analyzed experimental sites A, B, and C. (**A**–**C**).

**Figure 7:**
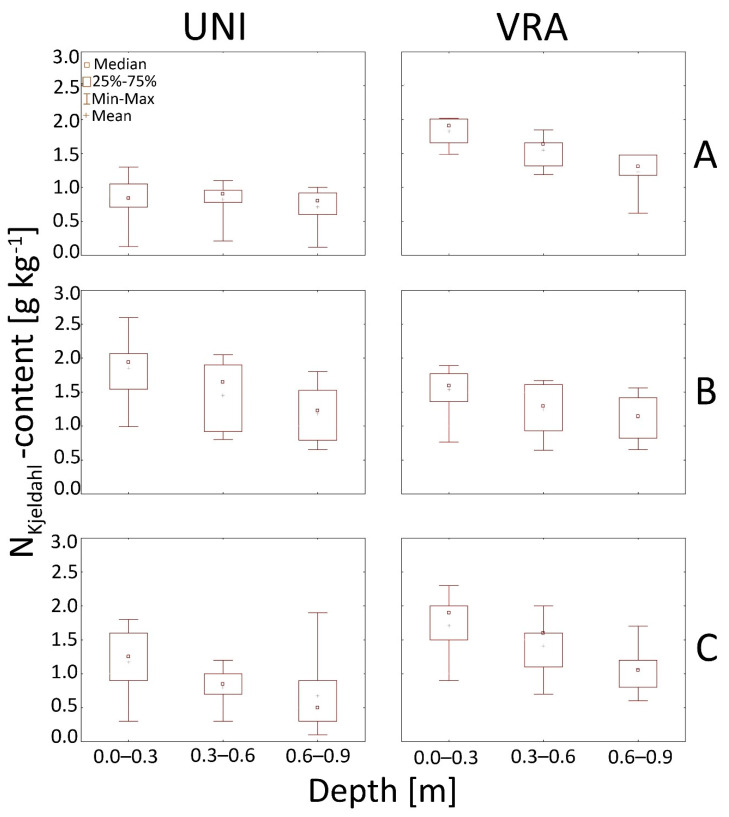
Box and whiskers plots presenting N_Kjeldahl_ content in the soil after uniform UNI and VRA fertilization at analyzed experimental sites A, B, and C. (**A**–**C**).

Our study also pointed out that nitrate as well as the content of the sum of ammonium and organic N in soils have a decreasing trend with depth both for UNI and VRA fertilization (Figure 6 and Figure 7).

The simple regression relationship between the concentration of specific N forms in soil and the soil depth were presented using mathematical equations (Table 5). 

Assuming a linear relationship between the concentration of N forms and depth, the equations were presented on the basis of which it was possible to determine the concentration of a selected form of N at a selected depth. Using the regression equations, it was calculated at what depth below the surface the concentration of N forms from fertilizers would be present in soil in trace amounts (concentration almost equal to 0 mg kg^−1^ for NH_4_-N and NO_3_-N or 0 g kg^−1^ for N_Kjeldahl_). 

As it has been shown in Table 3, it was calculated that the ammonium in soil of selected agricultural areas after UNI fertilization could be observed in trace amounts at the maximum depth 2.11 m, whereas the concentration of this form at the same fields after VRA fertilization could be observed at the maximum depth 1.16 m. The same was revealed for nitrate which could be observed in trace amount in soil after UNI fertilization at the depth of 1.46 m and at the depth of 1.28 m after VRA fertilization. The differences in calculated depths let us suppose that when the precise fertilization (adjusted to plant demands) was applied, the amount of unused N would be smaller and hence, the smaller amount of N forms would be transported to deeper parts of soil profile, and therefore to the groundwater system.

### 3.4. Content of N Forms in Soil vs. Soil Fractions

From all the data obtained, we can see that regardless the soil depth, after UNI fertilization, a positive correlation existed between ammonium and nitrate ions in soil. The same can be attributed to N_Kjeldahl_ and nitrate which showed a positive relationship. When comparing the relationships between specific soil fractions and the content of N forms, it can be seen that between sand fraction and nitrate as well as between clay fraction and nitrate, the positive correlation existed for all depths analyzed after UNI fertilization (Table 6). 

In case of VRA fertilization, it was observed that for all depths analyzed, the positive correlation existed between ammonium and N_Kjeldahl_. Moreover, there were positive relationships obtained between N_Kjeldahl_ and silt and clay fraction, respectively. It was also revealed that for each depth, the concentration of ammonium was positively correlated with the sum of silt fraction in soil (Table 7). That could be explained by the sorption of positively charged ion (ammonium) on negatively charged particles of soil. 

### 3.5. Spatial Fluctuations of N Content in Soil

**Figure 8:**
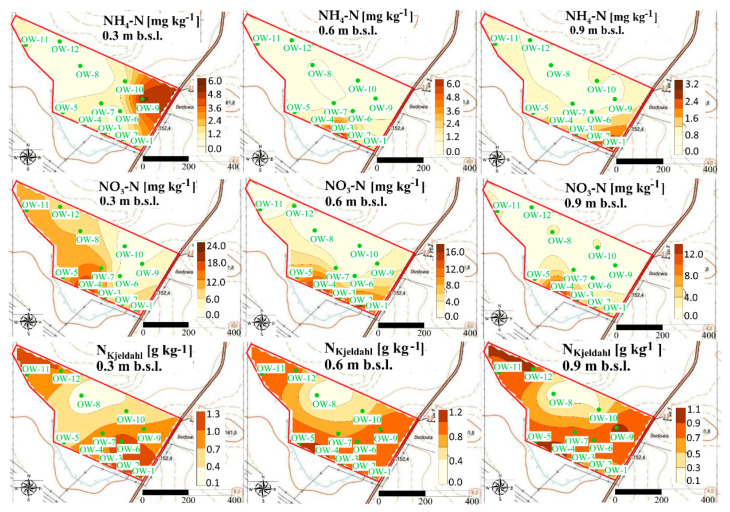
Spatial distribution of ammonium, nitrate, and N_Kjeldahl_ in soil at the A experimental site after UNI fertilization.

**Figure 9:**
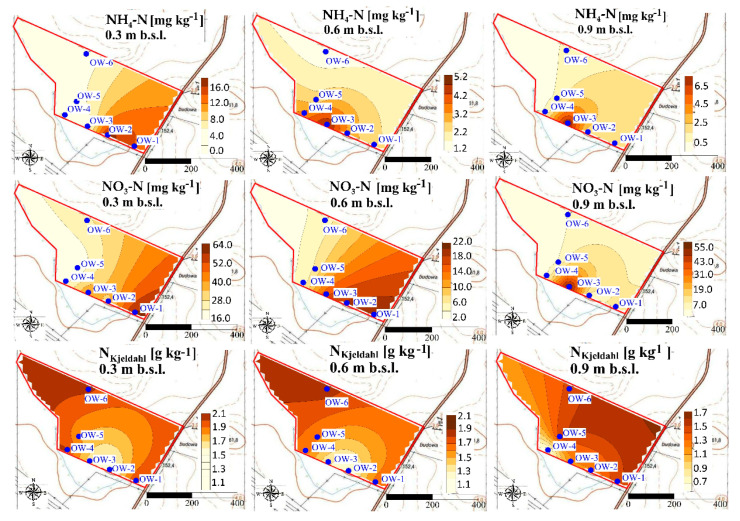
Spatial distribution of ammonium, nitrate, and N_Kjeldahl_ in soil at the A experimental site after VRA fertilization.

Our study revealed that concentrations of N forms show the spatial variability at each experimental site, after UNI and VRA fertilization. The highest concentrations observed at the A experimental site (Figure 8 and Figure 9) was lower than the average concentrations of ammonium measured for Polish arable soils [52]. 

It was also observed that the highest concentrations of nitrate were accumulated at the south part of the A experimental site, in locations of the lowest altitude (Figure 8 and Figure 9).

**Figure 10:**
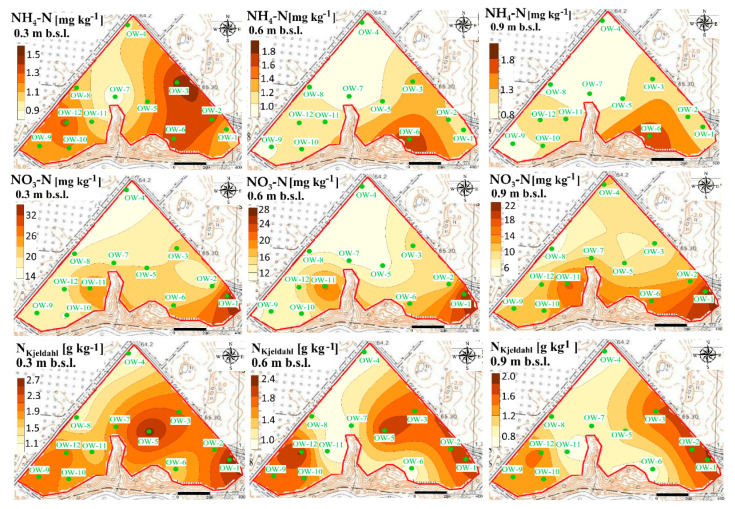
Spatial distribution of ammonium, nitrate, and N_Kjeldahl_ in soil at the B experimental site after UNI fertilization.

**Figure 11:**
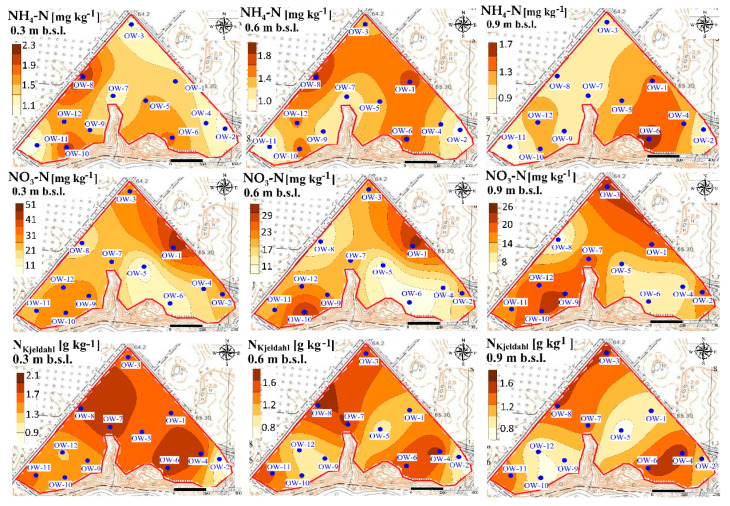
Spatial distribution of ammonium, nitrate, and N_Kjeldahl_ in soil at the B experimental site after VRA fertilization.

The fluctuations of N forms are also clearly visible at the B experimental site (Figure 10 and Figure 11). 

The greatest concentrations of ammonium at that site were observed after UNI fertilization in the vicinity of the sampling point OW-3 at the depth of 0.0–0.30 m (Figure 10 and Figure 11). 

The accumulation of ammonium was observed at the depth of 0.30–0.60 m and 0.60–0.90 m in the vicinity of the sampling point OW-6. 

**Figure 12:**
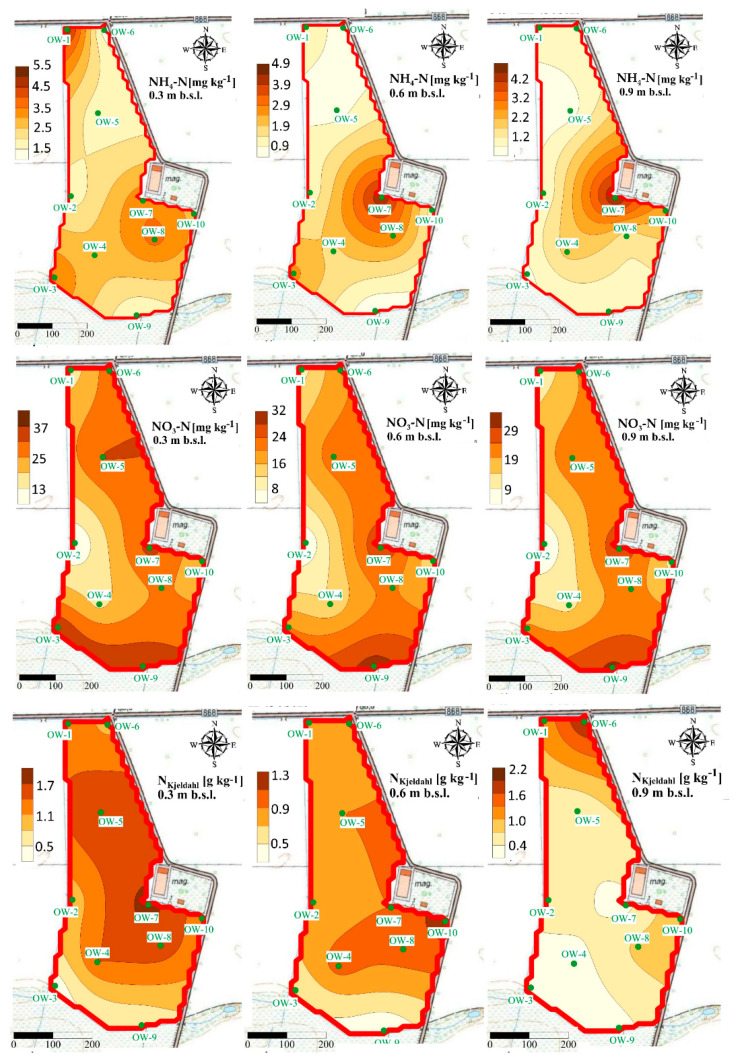
Spatial distribution of ammonium, nitrate, and N_Kjeldahl_ in soil at the C experimental after UNI fertilization.

It was also revealed that increased concentrations of nitrate after UNI fertilization were observed in the vicinity of sampling point OW-1, at each of the analyzed depths, which is slightly different after VRA fertilization (Figure 11). The content of the sum of ammonium and organic N and their hot-spots are distributed non-uniformly at the B experimental site. The greatest concentrations of ammonium at the C experimental site were observed in the vicinity of sampling point OW-7, after UNI fertilization (Figure 12).

**Figure 13:**
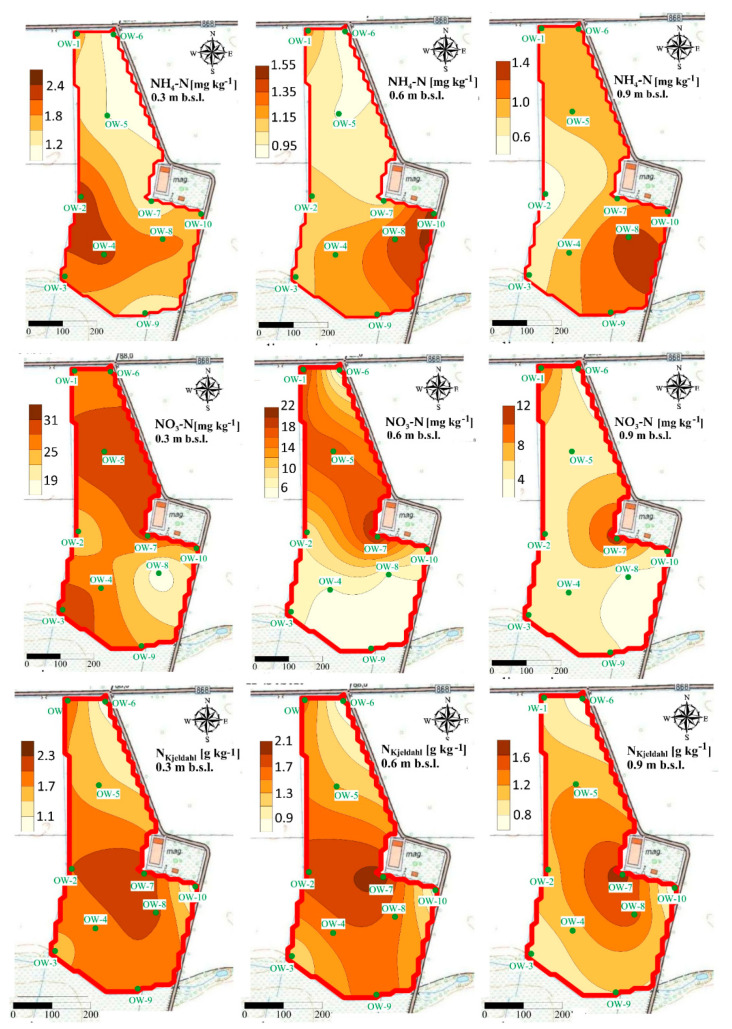
Spatial distribution of ammonium, nitrate, and N_Kjeldahl_ in soil at the C experimental site after VRA fertilization.

No evidence for this tendency can be observed at this site after VRA fertilization. A local increase in nitrate concentrations is observed at the C experimental site, both after UNI and VRA fertilization. The greatest concentrations of the sum of ammonium and organic N were observed in the vicinity of the sample point OW-7 and OW-8, both after UNI and VRA fertilization (Figure 12 and Figure 13). A deviation from this was observed at the depth of 0.60–0.90 m, after UNI fertilization, as the greatest N_Kjeldahl_ concentration was measured in the vicinity of the sampling point OW-6 (north part of the study site). The occurrence of elevated N concentrations should be treated as incidental and can be explained by local fluctuations in weather conditions.

## 4. Discussion

### 4.1. Typical Concentrations of N in Soil

According to the Monitoring of the Chemistry of Polish Arable Soils [52], the content of total N in soil is in the range of 400–4100 mg kg^−1^, while its average content is 1100 mg kg^−1^. In mineral soils, the N content is in the range of 200–4000 mg kg^−1^, while in organic soils, it can be up to 14,000 mg kg^−1^ [53]. It was also reported that minimum concentrations of nitrate in arable soils are at a level lower than 1 mg kg^−1^, whereas the maximum and median concentrations of nitrate in agricultural soils are equal to 110.58 and 5.42 mg kg^−1^, respectively. For comparison, Lee et al. [54] reported that the median concentration of nitrate measured in agricultural soils was 3 mg kg^−1^. The concentrations of nitrate measured at the analyzed sites were slightly higher than those measured for Polish arable soils. Monitoring data [55] showed that the minimum concentration of ammonium in agricultural soils was at the level of 0.43 mg kg^−1^, whereas the maximum concentration of this component was at the level of 42.6 mg kg^−1^. It can be stated that for agricultural sites presented in this study, the concentrations of ammonium were lower than the average concentrations measured for Polish agricultural soils. According to Lee et al. [54], the median concentration of ammonium in agricultural soils was equal to 4 mg kg^−1^, whereas in Lithuanian agricultural lands, concentrations of ammonium were observed at the level of 1 mg kg^−1^ at the depth of 0.00–0.30 m; 0.84 mg kg^−1^ at the depth of 0.30–0.60 m; and 0.84 mg kg^−1^ at the depth of 0.60–0.90 m. Nitrate concentrations measured in Lithuanian soils were equal to 6.45 mg kg^−1^ at the depth of 0.00–0.30 m; 4.19 mg kg^−1^ at the depth of 0.30–0.60 m, and 2.86 mg kg^−1^ at the depth of 0.60–0.90 m [56]. For comparison, Długosz and Piotrowska-Długosz [57] revealed that the average concentrations of the total N, nitrate, and ammonium in the soils of northwestern Poland were 1.99 g kg^−1^, 17.1 mg kg^−1^ and 11.1 mg kg^−1^, respectively. Literature findings indicate that proportions between ammonium and nitrate in soils show a specific tendency. Yan et al. [58] presented, based on their field plot experiment, that the content of ammonium was markedly lower than nitrate in the soil solution. In our study, it was revealed that at the analyzed experimental sites, the concentrations of ammonium are lower than nitrates (from 2 to 17 times at corresponding depths). In contrast to our findings, Sądej and Przekwas [59] revealed that ammonium dominated over nitrate in soils. Similar results were presented by Arbačauskas et al. [56] who documented that concentrations of nitrate also have a decreasing tendency through depth. Similar tendencies were also presented in our study. The comparison of measured concentrations of N forms with ranges of N form concentrations observed in different regions of the world let us state that analyzed experimental sites are comparable, in terms of N forms content, with other agricultural regions fertilized with the use of N fertilizers.

Keeping in mind that at a given N dose and under identified soil conditions N forms were observable in the examined concentration range may be an indication whether the doses of fertilizers used in the traditional system are adequate and there was no excessive accumulation of N in soil, or whether the application was excessive and unused N forms were sorbed in the soil or leach out, which could be also indicated by increased nitrate concentrations at greater depths of the soil profile.

### 4.2. Effect of Soil Type on N Forms Migration

Several authors reported that clay fractions can retain ammonium because of negative charge on their particles [60,61]. Literature findings indicated also that coarser soils are characterized by smaller retention than finer soils, which allows for faster leaching of nitrate into groundwater [62]. Moreover, the positive relationships between silt-clay content in soil and total N was proved [63].

It is known that nitrate is an ion that can migrate with water flow. Water does not flow easily in clayey layers and, as a result, nitrate is not freely leached to the groundwater system. Some scientific research proved that N losses by leaching were in the range from less than 10 to 30%, but it was also emphasized that leaching of N forms from coarse-textured soils may be greater than 30% [16].

It was also reported that nitrate is lost from clayey soils mainly by denitrification, which has an impact on the reduction of nitrate amount that can be released to groundwater. In this aspect, Arbačauskas et al. [56] revealed that the concentrations of nitrate in light-textured soils were lower in comparison with heavier textured soils. 

Ibrahim et al. [64] indicated that the clay had a positive direct effect on the N content, what can be attributed to the sorption phenomena. Then, it is also important that besides the sorption abilities, the retention of N forms in soil is also related to the form of clay fraction [65]. Generally, it was confirmed that organic matter in the fine clay fraction contained more N than coarse clay. According to Alamgir [66], the clay fraction has a high sorption capacity due to its specific surface area, chemical and mechanical stability, layered structure, and high cation exchange capacity. In contrast to clay soils, sandy soils are characterized by a lower sorption capacity and larger pore size, which makes them ineffective in retaining pollution. On this basis, Xu et al. [67] concluded that the total N in soil had a positive correlation with silt-clay fraction which has the greater ability to retain N ions migrating through the soil profile. What is also important, the proportion of clay and silt fraction in soils may have a great impact on the crop yield [68]. Due to this, it was revealed that the yield from soils represented by sandy loams was almost 30% higher than that from loamy sands, and the highest yield was reported for silty soils. 

Regarding literature findings reported above, we can say that at analyzed experimental sites, the vertical movement of N forms through the soil profile was successively limited by the occurrence of cohesive soils in the top parts of analyzed soil profiles. Because of the dominance of silty loams, and loams, no evidence of leaching N forms (no excessive concentrations) was observed at tested agricultural fields. Moreover, the lack of excessive concentrations of ammonium in deeper parts of the soil profile pointed out that existing soil conditions are unfavorable for the biochemical reduction of nitrate to ammonium.

### 4.3. Accumulation of N in Soil

Some scientific studies revealed that the excessive application of N fertilizers has a significant impact on the residual nitrate in soil, which can be seen by the accumulation of this ion in deeper soil layers [69]. The same was proved by Yan et al. [58], who indicated that excessive N application may lead to increased nitrate accumulation in the lower soil layers. The greater accumulation of N forms in soils can be observed more frequently in heavy texture soils (loams and clays) than in light texture soils (sands, sandy loams) [56,70]. Due to these facts and based on our findings presented in this study, we can state that the lack of accumulation of nitrate ions in deeper parts of analyzed soil profiles indicates that the N is applied suitably to the current plant demands. It was also revealed that lower concentrations of ammonium at the B experimental site can be attributed to the occurrence of sandy loams which have lower possibilities to retain N ions than heavier soils occurring at the A and C experimental sites.

The differences between N concentrations and accumulation in soil can be also attributed to contamination. In this regard, Uwah et al. [71] stated that significant differences (*p* < 0.05) between nitrate concentrations in soil samples and their corresponding levels in the control samples might be attributed to possible pollution resulting from an excessive use of agro-chemicals, and wastewater soil irrigation, and also can be attributed to the environmental conditions pertinent in the analyzed sites.

### 4.4. Decrease of N Form Concentration through the Depth

In the majority of cases it is not surprising that the decrease of N form concentrations with soil depth is observed, but without any analysis performed, the negative correlation between concentration of all N forms and soil depth should not be taken for granted. Regardless of the results obtained in this study, it is worth noting that the negative correlation between all N forms and depth is not always valid because sometimes totally opposite trends may be observed and they were also given by nature.

We may say that the correlation between N forms and depth is rather related to the specific study area and may be different for different sites. These observations can be also confirmed by the scientific research presented in the literature. For example, Bahmani et al. [60] reported that the content of ammonium decreases with the depth, and the concentration of ammonium is much lower than nitrate. Specifically, the decrease of ammonium through the depth can be explained by the nitrification process in the upper layers in which N in the form of ammonium is converted to nitrate. The same was proved by Yan et al. [58] who stated that ammonium derived from soil and fertilizers is rapidly transformed into nitrate through nitrification, resulting in lower ammonium and higher nitrate concentrations in the soil profile. The higher content of nitrate than ammonium in the same soil layer was also observed by Sądej and Przekwas [59] and Xu et al. [67]. The mentioned authors also reported that the gradual decrease of the total N content with soil depth can be attributed to N in organic matter. The decreasing trend of changes in N forms concentrations was also revealed in our study and can be explained by mentioned phenomenon.

### 4.5. Spatial Variability of N Forms Concentrations

Our study revealed that the fluctuations of N forms concentrations are clearly visible, but no patterns in their variability can be assessed as well as no tendency in occurring hot-spots of N concentrations can be indicated globally for analyzed fields. The concentrations of ammonium and nitrate change spatially over time and hot-spots of N forms may occur irregularly in time, depending on precipitation or N uptake by plants [72]. The spatial independence of N forms in soils was also reported by Hope et al. [73] and Spohn et al. [74] who pointed out that the contents of N forms (ammonium and nitrate) show high dynamics and variability. The random spatial variations and distributions of N forms in soils can be also attributed to differences in physical, chemical, and biological processes taking place in soils [75]. The soil moisture can be also treated as the factor determining the spatial distribution of nitrate which can be accumulated in dry soils due to reduced leaching [76]. The differences in the total N, ammonium, and nitrate distributions in soils were also reported by Długosz and Piotrowska-Długosz [57] who pointed out that it is crucial to take into consideration the variability of soil characteristics (soil type, content of N forms) for the purpose of a proper soil management and better understanding of the N transformations in the ecosystem. Hence, the knowledge about spatio-temporal behavior and the variability of nutrients is also essential for the management within precision agriculture [77]. The knowledge related to the spatial distribution of N forms was also found to be significant when assessing the patterns of groundwater contamination in the vicinity of landfill sites [78].

Due to the fact that N forms are very susceptible to transformation, maps presenting their distribution should be prepared to track their fluctuations only in a very short-term period. The susceptibility of N forms to transformations should be also taken into consideration when preparing and analyzing maps of their spatial distribution [79]. In this aspect, the best practice would be to create N distribution maps for every season, before the formulation any guidelines concerning site management [79]. 

### 4.6. Premises of Sustainable Development Goals (SDGs) Implementation

**Figure 14:**
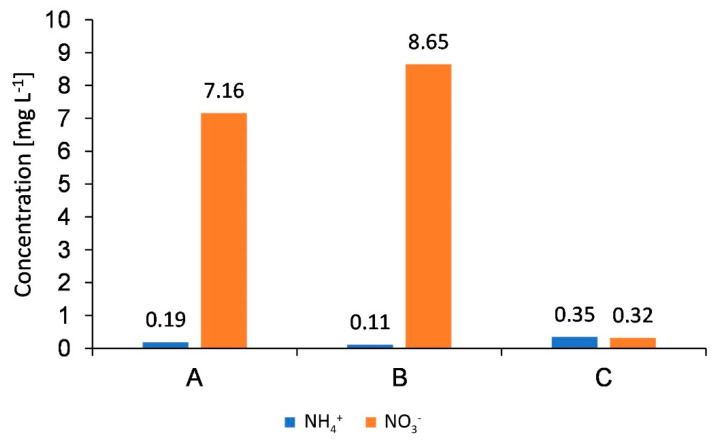
Concentrations of ammonium and nitrate ions in groundwater at the analyzed experimental sites: A, B, C [80,81].

The performed study addresses the Sustainable Development Goals (SDGs) adopted by the United Nations (UN). In most of the SDGs presented, soils play a crucial role as they are a link between the atmosphere–geosphere–hydrosphere–biosphere systems. The chemical and physical soil properties are also prerequisites for soil health. The performed study pointed out that proper agricultural management is required to ensure sustainable development and environmental safety. Soil vulnerability to anthropogenic threats should be studied as widespread contamination can threaten sustainable food production, clean water supply, climate change, and the health and quality of human life. To fulfill the SDGs, the integrated soil–crop system management is required. It would be possible by the application of the precision agriculture, which is a crucial way to maximize yield and minimize environmental impact by adapting agricultural management to local conditions and by optimizing nutrient application (N doses adjusted to real plant demands). Based on the performed research, we can conclude that the content of N forms in soils in connection with the soil data is relevant to optimize the application of N fertilizers in agricultural practice. Our outcomes strictly indicated that there are significant differences in N concentrations in soil, depending on the type of fertilization, soil properties, or soil depth. Significant differences were visible for the concentration of ammonium and nitrate, and the sum of ammonium and organic N (N_Kjedahl_) almost at each study site. We may also state that according to the precise fertilization, N is applied suitably to plant demands, which was proved by no accumulation of nitrate ions in lower parts of soil profiles. In this aspect, the movement of unused N forms to deeper parts of the soil, and therefore, to the groundwater system, is more limited due to the VRA fertilization (Figure 14). 

It was confirmed that the concentrations of N ions observed in groundwater samples (both nitrate and ammonium) indicate good chemical statuses. The average concentrations of nitrate measured in groundwater samples collected from piezometers located within the agricultural areas did not exceed the limit value of 10 mg L^−1^, which can be assigned to the first class of groundwater quality. Moreover, due to the precise fertilization, the average concentrations of ammonium in groundwater did not exceed the limit value of 0.5 mg L^−1^, characteristic for the first class of groundwater quality [80].

The results of performed study indicate that the recognition of soil and its properties is required for proper production of plant biomass and ensuring food safety, as stated in SDG 2. Moreover, it was indicated that the sorption capacities of soils can play a filtering role against contaminants and therefore protect the groundwater, as outlined in SDG 6.

### 4.7. Future Research Needs

Future research should be performed as a holistic investigation to control N losses and reduce agricultural costs while maintaining productivity. The joint actions should be taken among researchers, land managers, and policy makers to implement improved agricultural management practices that optimize agricultural production, minimize adverse effects on human health, and reduce environmental contamination. The approaches should also be put forward to strengthen environmental and economic sustainability to improve understanding soil N processes and plant uptake. 

The attempts should be made to improve the current knowledge related to the N use efficiency, especially with the special care to N recommendations, precision farming technologies, improving soil testing, and calculating nutrient budgets with advanced computer modeling tools and computer software.

The presented study should also be the starting point for future research focused on hydrogeological modeling aiming at the recognition of paths of N forms’ migration to the soil–water environment, taking into consideration information about their spatial diversity and influencing factors.

## 5. Conclusions

The presented research is a comprehensive study on N forms distribution in soils, with special attention given to different N forms and factors determining their content. Wide-scale analyses performed at selected study sites in Poland, and supported by the statistical evaluation of differences in N forms concentrations in soil, constitute an original contribution to the soil science and environmental management in agricultural areas. 

In accordance with the presented approach, management in agricultural practice should be based on the prediction of spatial variability of soil properties which allows to ensure proper application of fertilizers, resulting in the reduction of possible N losses. Nevertheless, the maps presenting spatial distribution of N forms in soils should be taken into consideration in the temporal aspect, concerning the fact that N forms are very susceptible to transformation due to physical, chemical, and biological processes. 

## Figures and Tables

**Table 1:** Summary of selected precision agriculture research carried out in Poland in 2015–2020.

No.	Objective	Key Results	Ref.
1	Presentation of the existing methods of micronutrient fertilization.	Precise fertilization techniques based on low-solubility fertilizers, coated fertilizers, bio-based, and nanofertilizers are a new trend in modern agriculture.	[16]
2	Identifying the diversity of soil profile cohesion on the basis of non-invasive measurement of the electrical conductivity.	Maps of spatial differentiation of electrical conductivity within the field for their further use in precision agriculture.	[17]
3	Concept of a circularly polarized antenna with partially reflecting surface (PRS) has been adopted for precision farming applications.	Designed antennas employed for point-to-point communication in systems of mobile devices or vehicles used under precision farming.	[18]
4	Creation of independent, multi-criteria models for the prediction of winter rapeseed yield.	Forecasting winter rapeseed yields using artificial neural networks makes it possible to obtain an accurate yield forecast before harvesting. The concept of neural modeling may contribute to sustainability by reducing the doses of mineral fertilizers.	[19]
5	Study on precision agriculture concept and application.	Obtaining data concerning spatial variability of soil and plants, discussion on remote sensing, and advanced digital technology application in precision agriculture.	[20]
6	Study on the use of remote sensing in precision agriculture.	Discussion on precision agriculture in aspect of steering of farm machinery, monitoring of biomass and crop yields, soil collection, doses of mineral fertilization.	[21]
7	Analysis of use of machine vision in modern agriculture.	Examples of the use of the CloverCam system, the WeedSeeker system, Robot RoniBob Amazone Bosch, autonomous robot Agrobob.	[22]
8	Presentation of the latest trends related to the digitization of agricultural processes.	Due to the process of digitization of agriculture, in the near future, resource management will be more effective, which will reduce the impact of farming and crops on the environment, supporting sustainable agriculture.	[23]
9	Estimation of potato yields.	Examples of use of remote sensing, vegetation indices, forecasting models, artificial neural networks, and image analysis methods in yields prediction.	[24]
10	Analysis of the application of a high precision positioning system ASG-EUPOS and its service NAWGEO for agricultural machines positioning.	Field tests show usefulness of the ASG-EUPOS network and its VRS NAWGEO service for precise positioning of agricultural machinery in dynamic conditions. The obtained data can be used to create numerical models of fields on-line, for example, in selective cereals harvesting technology.	[25]
11	Identification of soil properties in different weather conditions during the growing season and mapping soil properties and crop yields using inverse distance weighting.	Geostatistical analysis is a useful tool to determine spatial interrelationships of crop yield and soil properties in the scale of agricultural field.	[26]
12	Analysis of vegetation indices used to carry out a precise and non-invasive assessment of plants condition.	Creation of vegetation indices maps (NDVI, GNDVI, SAVI). It was concluded that proper interpretation of the obtained indicators will allow for the preparation of fertilizer applications.	[27]
13	Analysis of precision agriculture methods and application.	The application of the principles of precision farming has a positive effect on reducing contamination. Discussion on variable rate application of fertilizers.	[28]
14	Evaluation of the sensitivity of sensor-based N-rate prescriptions for winter wheat to selection of sample strips for AOS calibration.	The choice of a sample strip for AOS calibration could significantly affect sensor variable N rates prescribed for winter wheat.	[29]
15	Presentation of innovative solutions for plant production in Poland.	Innovative technologies in agricultural production may reduce the negative impact of climate change.	[30]
16	Evaluation of the precision agriculture technology on the territory of Podlaskie Voivodeship in Poland.	Only 10% of farmers use the positioning system and only 8% of the surveyed farmers apply the system for guiding agricultural machines. In addition, 14% of the investigated farmers use the system of parallel guiding.	[31]
17	Analysis of techniques for photographing and scanning crops from drones and creating field maps.	Information was obtained that could be read by the automatic control systems of machines used for fertilization and plant protection, as well as for harvesting crops. It was concluded that the use of drones in agriculture contributes to economic results.	[32]
18	Evaluation of the performance of active optical sensor (AOS) by determination of grain yield, N fertilizer use, grain protein content, N use efficiency, and N balance, utilizing a built-in algorithm for variable N rate fertilization of winter wheat.	Implementation of AOS for variable N application would minimize N surplus in areas of low productivity and improve the sustainability of N management.	[33]
19	Analysis of the use of remote sensing data in crop yield forecasting, assessing nutritional requirements of plants and nutrient content in soil, determining plant water demand and weed control.	Use of remote sensing to determine fertilization needs of plants based on the nutrient content of crops and soils helps to increase yields and improve the crop profitability.	[34]
20	Presentation of the evolutionary transition of conventional systems of agricultural activity to environmentally sustainable systems, integrated with the rural environment.	The concept of the organization of the agricultural precision production system in selected (certified) ecological farms was presented.	[35]
21	Evaluation of the soil texture prediction accuracy and the main criteria by which prediction accuracy is estimated.	All soil texture fractions were predicted with similar accuracy, using inverse distance weighting, radial basis function, ordinary kriging, and ordinary cokriging.	[36]

**Table 2:** Characteristics of the study sites and fertilization.

Characteristics	Experimental Site		
	A	B	C
Area	20 km^2^	40 km^2^	20 km^2^
Climate classification ^1^	Cfb	Dfb	Cfb
Average annual temperature	9.5 °C	8.2 °C	9.0 °C
Total annual precipitation	646	721	612
First sampling/fertilization type	May/UNI	May/UNI	May/UNI
Days from UNI fertilization to soil sampling	46	33	25
Second sampling/fertilization	September/VRA	November/VRA	September/VR
Days from VRA fertilization to soil sampling	136	159	126
Fertilizer used—UNI	32% ammonium nitrate	32% urea ammonium nitrate solution	24% N and 15% S Sulfan
Fertilizer used—VRA	32% ammonium nitrate	32% ammonium nitrate	34% ammonium nitrate
UNI fertilization doses	74 kg N ha^−1^	80 kg N ha^−1^	60 kg N ha^−1^
VRA fertilization doses	30–70 kg N ha^−1^	40–90 kg N ha^−1^	55–105 kg N ha^−1^

^1^ according to the Köppen–Geiger system [44].

**Table 3:** Summary of the statistical analysis of differences in the measured content of N forms in soils after uniform (UNI) and spatially variable (VRA) fertilization.

N Form	Field	Test	*p* Value	Mean Concentration ^1^ ± SD ^2^	
				UNI	VRA
NH_4_-N	A	Mann–Whitney U	0.000867 ^3^	1.49 ± 1.38	4.59 ± 4.46
	B	Mann–Whitney U	0.018582 ^3^	1.10 ± 0.22	1.29 ± 0.35
	C	Mann–Whitney U	0.083671	1.23 ± 0.44	1.96 ± 1.33
NO_3_-N	A	Mann–Whitney U	0.002866 ^3^	5.49 ± 5.40	18.78 ± 18.23
	B	Student t	0.027702 ^3^	15.46 ± 5.88	19.34 ± 8.53
	C	Mann–Whitney U	0.079757	13.81 ± 10.03	18.08 ± 10.02
N_Kjeldahl_	A	Mann–Whitney U	0.000000 ^3^	0.79 ± 0.28	1.54 ± 0.36
	B	Mann–Whitney U	0.068934	1.49 ± 0.54	1.30 ± 0.38
	C	Student t	0.000140 ^3^	1.39 ± 0.49	0.88 ± 0.48

^1^ expressed in mg kg^−1^ for NH_4_-N and NO_3_-N and g kg^−1^ for N_Kjeldahl_, ^2^ standard deviation, ^3^ values indicate concentrations statistically different.

**Table 4:** Correlation between the content of N forms in soil profile and soil depth, calculated separately for A, B, C experimental sites.

Depth	**NH_4_-N**	**NO_3_-N**	**N_Kjeldahl_**	**Fertilization**	**Experimental Site**
	−0.16	−0.32	−0.17	UNI	A
	−0.59 ^1^	−0.47	−0.71 ^1^	VRA	
	−0.24	−0.65 ^1^	−0.52 ^1^	UNI	B
	−0.38 ^1^	−0.44 ^1^	−0.46 ^1^	VRA	
	−0.38 ^1^	−0.58 ^1^	−0.43 ^1^	UNI	C
	−0.69 ^1^	−0.85 ^1^	−0.55 ^1^	VRA	

^1^ correlation significant at the level of *p* < 0.05.

**Table 5:** Concentration of N form as a function of soil depth.

Fertilization	N Form	Equation	Maximum Depth D [m]
UNI	NH_4_-N	NH_4_-N = 2.0787 − 0.9813 × D	2.11
UNI	NO_3_-N	NO_3_-N = 21.511 − 14.67 × D	1.46
UNI	N_Kjeldahl_	N_Kjeldahl_ = 1.4910 − 0.7103 × D	2.09
VRA	NH_4_-N	NH_4_-N = 4.4291 − 3.786 × D	1.16
VRA	NO_3_-N	NO_3_-N = 32.189 − 25.14 × D	1.28
VRA	N_Kjeldahl_	N_Kjedahl_ = 1.8387 − 0.7958 × D	2.31

**Table 6:** Spearman correlation between the content of N forms and soil fractions at different depths; after uniform fertilization (UNI).

Depth	Parameter	NH_4_-N	N-NO_3_	N_Kjeldahl_
0.00–0.30 m b.s.l.	NH_4_-N	1.00	0.35	−0.10
	NO_3_-N	0.35	1.00	0.35 ^1^
	N_Kjeldahl_	−0.10	0.35 ^1^	1.00
	Sa	−0.04	0.39 ^1^	0.68 ^1^
	Si	0.02	−0.47 ^1^	−0.67 ^1^
	Cl	0.45 ^1^	0.26	−0.12
	Si + Cl	0.05	−0.39 ^1^	−0.69 ^1^
0.30–0.60 m b.s.l.	NH_4_-N	1.00	0.31	0.21
	NO_3_-N	0.31	1.00	0.16
	N_Kjeldahl_	0.21	0.16	1.00
	Sa	0.00	0.36 ^1^	0.57 ^1^
	Si	−0.11	−0.44 ^1^	−0.57 ^1^
	Cl	0.24	0.29	−0.10
	Si + Cl	−0.01	−0.35	−0.57 ^1^
0.60–0.90 m b.s.l.	NH_4_-N	1.00	0.42 ^1^	0.13
	NO_3_-N	0.42 ^1^	1.00	0.11
	N_Kjeldahl_	0.13	0.11	1.00
	Sa	0.19	0.46 ^1^	0.39 ^1^
	Si	−0.15	−0.48 ^1^	−0.41 ^1^
	Cl	−0.04	0.15	0.00
	Si + Cl	−0.15	−0.38 ^1^	−0.38 ^1^

^1^ correlation significant at the level of *p* < 0.05.

**Table 7:** Spearman correlation between the content of N forms and soil fractions at different depths; after variable fertilization (VRA).

Depth	Parameter	N-NH_4_	N-NO_3_	N_Kjeldahl_
0.00–0.30 m b.s.l.	NH_4_-N	1.00	0.09	0.30
	NO_3_-N	0.09	1.00	−0.21
	N_Kjeldahl_	0.30	−0.21	1.00
	Sa	−0.56 ^1^	−0.09	−0.35
	Si	0.58 ^1^	0.15	0.32
	Cl	−0.09	−0.24	0.26
	Si + Cl	0.55 ^1^	0.08	0.33
0.30–0.60 m b.s.l.	NH_4_-N	1.00	−0.06	0.11
	NO_3_-N	−0.06	1.00	−0.08
	N_Kjeldahl_	0.11	−0.08	1.00
	Sa	−0.20	0.02	−0.32
	Si	0.16	−0.05	0.24
	Cl	−0.05	0.12	0.26
	Si + Cl	0.21	−0.01	0.30
0.60–0.90 m b.s.l.	NH_4_-N	1.00	0.41 ^1^	0.18
	NO_3_-N	0.41 ^1^	1.00	−0.05
	N_Kjeldahl_	0.18	−0.05	1.00
	Sa	−0.19	0.16	−0.31
	Si	0.23	−0.04	0.31
	Cl	0.09	0.06	0.18
	Si + Cl	0.28	−0.05	0.28

^1^ correlation significant at the level of *p* < 0.05.

## Data Availability

Data sharing is not applicable to this article.
